# Piezoelectric Biocomposites for Bone Grafting in Dentistry

**DOI:** 10.3390/polym15112446

**Published:** 2023-05-25

**Authors:** Cristina Rodica Dumitrescu, Ionela Andreea Neacsu, Roxana Trusca, Roxana Cristina Popescu, Iuliana Raut, Mariana Constantin, Ecaterina Andronescu

**Affiliations:** 1 Department of Impact of Build Environment and Nanomaterials, National Institute for Research and Development in Environmental Protection, 294 Splaiul Independenței Blv, 060031 Bucharest, Romania; cristinadumitrescu0@gmail.com; 2 Department of Science and Engineering of Oxide Materials and Nanomaterials, Faculty of Chemical Engineering and Biotechnology, University Politehnica of Bucharest, 060042 Bucharest, Romania; ecaterina.andronescu@upb.ro; 3 National Research Center for Micro and Nanomaterials, University Politehnica of Bucharest, 060042 Bucharest, Romania; roxanatrusca@gmail.com; 4 Academy of Romanian Scientists, Splaiul Independentei Street No. 54, 011061 Bucharest, Romania; 5 Department of Bioengineering and Biotechnology, Faculty of Medical Engineering, University Politehnica of Bucharest, 060042 Bucharest, Romania; roxana.popescu1108@upb.ro; 6 Department of Life and Environmental Physics, National Institute for Research & Development “Horia Hulubei”, 30 Reactorului Street, 077125 Magurele, Romania; 7 National Institute for Research & Development in Chemistry and Petrochemistry- ICECHIM, Splaiul Independentei Street No. 202, 060021 Bucharest, Romania; raut.iuliana@icechim.ro (I.R.); marriconstantin@yahoo.com (M.C.); 8 National Research Center for Food Safety, University Politehnica of Bucharest, 060042 Bucharest, Romania

**Keywords:** bio-piezocomposites, piezoelectric antibacterial effect, hydroxyapatite, alkali niobate, chitosan, tissue engineering, bone grafting composite

## Abstract

In this research, Hydroxyapatite—Potassium, Sodium Niobate—Chitosan (HA-KNN-CSL) biocomposites were synthesized, both as hydrogel and ultra-porous scaffolds, to offer two commonly used alternatives to biomaterials in dental clinical practice. The biocomposites were obtained by varying the content of low deacetylated chitosan as matrix phase, mesoporous hydroxyapatite nano-powder, and potassium–sodium niobate (K_0.47_Na_0.53_NbO_3_) sub-micron-sized powder. The resulting materials were characterized from physical, morpho-structural, and in vitro biological points of view. The porous scaffolds were obtained by freeze-drying the composite hydrogels and had a specific surface area of 18.4—24 m^2^/g and a strong ability to retain fluid. Chitosan degradation was studied for 7 and 28 days of immersion in simulated body fluid without enzymatic presence. All synthesized compositions proved to be biocompatible in contact with osteoblast-like MG-63 cells and showed antibacterial effects. The best antibacterial effect was shown by the 10HA-90KNN-CSL hydrogel composition against *Staphylococcus aureus* and the fungal strain *Candida albicans*, while a weaker effect was observed for the dry scaffold.

## 1. Introduction

In recent years, bone addition biomaterials utilization in dentistry has been constantly growing, as evidenced by a huge research interest in this field [1]. In this context, piezoelectric smart materials have gained considerable attention in the biomedical community for their ability to change their electric properties as a response to external mechanical stimuli, such as those happening during mastication [2]. State-of-the-art biomaterials used in dentistry as bone substitutes must offer progressive benefits, in addition to excellent biocompatibility and bone mimicry in terms of both composition and structure [3]. Just to list a few of these requirements, stimulating osteogenic effects, antibacterial effects, and hemostatic or anti-inflammatory properties have shown considerable importance [4,5]. From the structural and compositional mimicry point of view, collagen/hydroxyapatite-based bone substitutes seem to be suitable candidates [6,7,8]. However, only by using bone-mimetic composition, additional osteopromoting and antibacterial properties cannot be achieved. Nevertheless, this can be achieved by piezoelectric stimulation [4,9,10,11]. The weak piezoelectric effect of biopolymers is not enough to activate the osteoblasts, but stimulated piezoceramics could develop enough polarization for improved bio-signaling or calcium channels opening, charged biomolecule interaction, and pH-dependent processes [12,13,14,15]. The amino-polysaccharide chitosan can be an alternative material for the preparation of tissue-engineered scaffolds due to its biodegradability, adsorption properties, and ability to support cell differentiation [16]. The mechanical properties of chitosan can be controlled by altering its crystallinity, microstructure, molecular weight, and degree of deacetylation, respectively [17,18]. However, chitosan’s low degree of deacetylation can have a few shortcomings, such as allergic reactions and low solubility [19]. It has been reported that chitosan promotes the growth and differentiation of osteoblasts in cell culture [19,20,21], possesses an antibacterial effect [4,22], has cholesterol-lowering properties [23], and acts as a hemostatic agent [24]. Nevertheless, chitosan itself is not osteoconductive and does not have enough bone formation ability, so combining the strengths of different materials would minimize their drawbacks [25,26]. In this regard, hydroxyapatite (HA) and tri-calcium phosphate (β-TCP) have been widely applied in bone tissue engineering due to their interesting properties, mainly those of tissue-mimicking and biocompatibility, adhesivity, and osteoconductivity, but in some cases, they are being resorbed before completing the new bone formation [27,28]. Showing a versatile composition and structure, biogenic resourced HA, especially with amorphous phase content [29,30] or nano-powders, leads to endothelial cell growth and angiogenesis [31,32,33], can regulate T cell immune response [34], and can induce early dental caries remineralization [35,36]. Piezoelectric smart biomaterials like barium titanate (BT), zinc oxide (ZnO), and potassium–sodium niobate (KNN) can be applied in theragnostic or tissue engineering, since these powders can be loaded with both therapeutic molecules [35,37,38,39] and, e.g., contrast agents [40,41,42], and could release the active substances at the site being activated by external energy (e.g., ultrasound) [43] or mechanical stimulation [44,45,46]. Therefore, it remains a challenge to induce theragnostic effects, but exploiting the piezoelectric properties of materials could be especially useful in rehabilitating tissue parts damaged through congenital defects, trauma, or carcinogenic diseases [9]. For bone tissue applications, scaffolds based on piezoelectric materials are an attractive choice, as they induce various phenotypic and genetic alterations to the subjected cells, which accelerate the repairing processes [47,48,49].

Nowadays, the classical materials used in dentistry are bone grafts, showing accepted moderated risks and modest results [50]; however, the osteogenic processes and healing time can be improved with the significant contribution of KNN-based scaffolds or powders, because of mastication-induced piezoelectric effects [51,52]. In addition, KNN-based biocomposites can also exhibit antibacterial effects. In this case, mastication is the mechanical tension that will stimulate the piezoelectric process of KNN powder from the chitosan matrix. When mechanically stimulated, the granules start polarization and induce the antibacterial effect. The adhesion of bacteria on negatively polarized surfaces is expected to be reduced due to electrostatic repulsion between the charge on the bacterial membrane (negative charge) and the material surface [53,54,55].

In this paper, a new generation of bioactive piezoelectric biocomposites obtained in the forms of porous scaffolds and hydrogels, adapted for easy implementation in dental grafting techniques, were pursued. The composites were compositionally and morpho-structurally designed to fulfill the requirements for synthetic bone substituents with improved mineralization and cellular signaling processes. Hence, the proposed composite material aims to mimic the bone from a compositional point of view by combining chitosan—a natural polymer likewise collagen and hydroxyapatite—with the inorganic phase of the natural bone, as well as from a functional point of view, by adding KNN—a powder with a strong piezoresponse. Chitosan belongs to the cationic kind due to its amino functional group specific to the aceto-amino polysaccharide range. On the other hand, the piezoelectric micrometric grains of KNN are positively charged on a face and negatively charged on the opposite one after mechanical stress. When designing the composition and microstructure of the composite, an “electrical involvement” between long polysaccharide chains and KNN grains was desired. Hence, the composite matrix (CS) should self-degrade in the physiological environment to perform its functional features (i.e., hemostatic, and antibacterial activity), while KNN grains should be polarized by a mastication process to exhibit antibacterial and osteoinductive behavior. By varying the piezoelectric powder content, differences in the antibacterial and antifungal response have been observed in the absence of external electric stimulation. Moreover, these proposed composites manifest in vitro biocompatibility and high cell metabolic activity, which can also be attributed to the piezoelectric component.

## 2. Materials and Methods

### 2.1. Biocomposite Synthesis

#### 2.1.1. Hydroxyapatite Nano-Powder (HA)

The powder was synthesized from natural sources: eggshell calcinated at 800 °C for 3 h and coprecipitated using di-base ammonium phosphate as precursors and maturated using a hydrothermal microwave-assisted method using the process parameters: 10.8 bars pressure, a temperature of 200 °C for an entire treatment cycle of 60 min. The hydroxyapatite powder thus obtained (HA1) had nanoparticles with a rod-like morphology with average length and diameter of 77.29 nm and 21.74 nm, respectively, and high internal and surface mesoporosity (mean size of 3.3 ± 1.6 nm). The polycrystalline particles crystallized in monoclinic symmetry and had a high crystallinity (84%). The HA sample synthesis and characterization were detailed elsewhere [56].

#### 2.1.2. Potassium–Sodium Niobate Powder (KNN)

The powder was synthesized starting from a suspension with an alkaline (KOH + NaOH) = 10 M molar rate K/Na = 8 M/2 M in which the niobium pentoxide powder was stabilized by magnetic stirring for 45 min, followed by a hydrothermal microwave-assisted maturation for a dwelling time of 30 min at 250 °C and at high pressure (40 bars). The solid solution powder thus obtained had a molar fraction x = 0.47 (K_0.47_Na_0.53_NbO_3_), and submicronic polycrystalline particles (mean size 369 ± 19.63 nm) with cubic-like morphology. Measured in unconventional conditions (unsintered and unpolarized), the cylindrical specimen of KNN evidenced a piezoelectric coefficient d_33_ of 1.20 pC/N small frequency of 50 Hz. Details about potassium–sodium niobate solid solution (KNN) synthesis and powder characterization could be found in the reference [57].

#### 2.1.3. Chitosan Solution

For chitosan solution preparation, the following were used: chitosan powder with low molecular weight 50,000–100,000 Da, deacetylated degree (DD) 75–85%, Brookfield viscosity 20–300 cP (at 25 °C), and glacial acetic acid (≥99.7% purity) from Sigma-Aldrich Chemie GmbH—Schnelldorf, Germany and ultrapure water. A 2% *w/v* chitosan solution in 1.5% *v/v* aqueous acetic acid solution was prepared by continuous stirring for 3 h at room temperature until it became clear (CSL) [13].

#### 2.1.4. Composite Hydrogels and Porous Scaffolds Preparation

First, the HA and KNN powders were mixed in an agate mortar with a pestle for 15 min. The three samples described in Table 1 were obtained by embedding the powder mix (HA, KNN) in the chitosan solution using a magnetic stirrer for 24 h until the mixtures were clear and stable. The obtained compositions, as well as the sole chitosan solution, were cross-linked with aqueous glutaraldehyde 1 wt.%. After reticulation, the samples were repeatedly soaked in distilled water until a negative result was obtained on the Fehling test, to assure the removal of free glutaraldehyde.

The three compositions of hydrogels were frozen for 48 h at −10 °C, followed by a lyophilization cycle of 96 h in a vacuum (0.01 bar) at −75 °C. Therefore, the porous composite scaffolds (sponges) had the same compositions as the three gels synthesized and are further referenced as 90HA-10KNN-CSL, 10HA-90KNN-CSL, and 50HA-50KNN-CSL.

### 2.2. Biocomposite Characterization

#### 2.2.1. X-ray Diffraction

X-ray Diffraction (XRD) was performed using PANalytical Empyrean Spectrometer (Malvern PANalytical, Bruno, The Netherlands), operating in Bragg-Brentano configuration with Cu-Kα (λ = 1.5406 Å) with working voltage and current of 40 kV and 100 mA, respectively. The spectra were recorded between 100 < 2*θ* < 800 with a scan speed of 0.5°/min and a step size of 0.02°.

#### 2.2.2. IR-Spectra

Fourier-transform infrared spectroscopy (FTIR) spectra were recorded in the wavenumber range of 4000–500 cm^−1^, in increments of 1.928 cm^−1^, using a Nicolet iS50R spectrometer (Thermo Fisher, Waltham, MA, USA) in attenuated total reflection mode (ATR). Each spectrum was collected at room temperature at a resolution of 4 cm^−1^, and 32 samples were scanned between 4000 and 440 cm^−1^.

#### 2.2.3. Scanning Electron Microscopy (SEM)

A Quanta Inspect F scanning electron microscope (SEM) (Thermo Fisher, Eindhoven, The Netherlands) equipped with a field electron emission gun (FEG) and an EDS (Energy Dispersive Spectroscopy) detector were used. The technical parameters were an acceleration voltage of 30 KV and a point-to-point resolution of 1.2 nm. The histograms of samples were obtained from the statistical processing of images using the Image J software.

#### 2.2.4. BET (Brunauer, Emmett, Teller) Specific Surface Area

The BET specific surface area, pore volume, and pore radius determinations were performed on porous granular material obtained from lyophilized scaffolds using the NOVA 2200E BET Surface Area Analyzer (Quantachrome, Boynton Beach, FL, USA), and the BELMaster™ 7 software data acquisition system converted information to generate the absorption-desorption isotherms. The method uses nitrogen gas (N_2_) by varying its pressure between 0.001 and 1 atmosphere.

#### 2.2.5. Swelling Test and Degradation in SBF

The swelling test was performed using scaffold-type biocomposites and SBF as submerge medium. Each sample of composite scaffold (sponge) was weighed before immersion and after immersion; the duration of immersion was set at different time intervals: each 1–5 min, then at 60 min, 7, and 28 days. Equation (1) was used to determine the amount of liquid absorbed and the degree of swelling [58]:

S_Wi_ = (m_f i_ − m_d_)/m_d_ = m_water i/_m_d_ (1)

where: Swi is the swelling rate after i minutes of immersion; m_f i_is the sample weight after i minutes of immersion; m_d_ is the dry sample weight (before immersion); and m_water i_ = m_f i_ − m_d_

In vitro degradation assessment of composite sponges was also made by samples submerged in SBF and incubated in a thermostatic water bath at 36.5 °C for 7 and 28 days, respectively, simulating degradation conditions in physiological fluid substitute (SBF). The preparation of the SBF solution was carried out following the protocol described in the literature [59], with the composition at 1000 mL: ultra-pure water 750 mL, NaCl 7.996 g, NaHCO_3_ 0.350 g, KCl 0.224 g, K_2_HPO_4_·3H_2_O 0.228 g, MgCl_2_ 6H_2_O 0.305 g, HCl (1 M) 40 cm^3^, CaCl_2_ 0.278 g, Na_2_SO_4_ 0.071 g, (CH_2_OH)_3_CNH_2_ 6.057 g, and HCl (1M) 12 cm^3^ for pH adjustment at 7.35. After the incubation period ended, the fragments of composite scaffolds were dried at room temperature for 24 h.

#### 2.2.6. Cell Viability (MTT Assay)

*Cell culture*

To assess the biological effect of the samples, the MG-63 osteoblast-like cells (CLS, Eppelheim, Germany) were used. The cells were cultured in Dulbecco’s Modified Eagle Medium (DMEM, Biowest, Riverside, Newry and Mourne, UK) supplemented with 10% fetal bovine serum (FBS, Biowest), in standard conditions of temperature and humidity (37 ± 2 °C, 5 ± 1% CO_2_ and more than 90% humidity).

In the case of the sponge-like samples (10HA-90KNN-CSL, 90HA-10KNN-CSL, CSL) and gel samples (10HA-90KNN-CSLG, 90HA-10KNN-CSLG, CSLG) that were previously sterilized by gamma irradiation, direct contact test was applied. Therefore, 10^5^ cells in 500 µL were directly seeded into a twelve-well culture plate. The cells were allowed to attach for 24 h and afterward one sponge (cylindrical shape 6 × 4 mm) 30 µL of gel sample was added in each corresponding well, to be in direct contact with the previously seeded cells. Then, the samples were incubated in standard conditions of temperature and humidity for another 24 h, 72 h, and 7 days.

In the case of the nanoparticle samples (HA and KNN) that were previously sterilized by gamma irradiation, 5000 cells in 100 µL were directly seeded into ninety-six-well culture plates and allowed to attach for 24 h in standard conditions of temperature and humidity. Afterward, binary dilutions of the nanoparticle samples were prepared in a complete culture medium (0–200 µg/mL) by ultrasound dispersion. The culture medium in each well was replaced with nanoparticles containing medium and incubated for another 24 h, 72 h, respectively, for 7 days in standard conditions of temperature and humidity. Blank samples, meaning samples containing nanoparticles but no cells, were also prepared, to remove interferences.

Following this incubation time, the cellular morphology was assessed using optical microscopy imaging with no prior preparation of the cell samples.

*MTT Assay*

The cellular viability was quantitatively measured using the MTT tetrazolium-salt assay (Serva Electrophoresis GmbH, Heidelberg, Germany). For this, at the corresponding time point, the medium was removed and gently replaced with fresh culture medium containing 10% MTT solution (5 mg/mL in PBS). The cells were incubated for another 2 h in standard conditions, and afterward, the supernatant was replaced with DMSO to solubilize the grown formazan crystals. The absorbance corresponding to each sample was measured at 570 nm wavelength.

Control (untreated) samples were also prepared, and the absorbance measured for these samples was attributed to the value of 100%. All other samples’ viabilities were calculated by reporting their absorbance to control samples. The results were evaluated in conformity with “EN ISO 10993-5:2009 Tests for in vitro cytotoxicity”.

All experiments were performed in triplicate and the data were presented as mean ± SD. The statistical analysis was performed using a two-tailed Student’s test, where values of * *p ≤* 0.05, ** *p* ≤ 0.01, *** *p* ≤ 0.001 were considered statistically significant.

#### 2.2.7. Antibacterial and Antifungal Activity

For the evaluation of antimicrobial activity, the following strains of bacteria were used: *Staphylococcus aureus*, ATCC 25,923 (*S. aureus*), *Escherichia coli*, ATCC 25,922 (*E. coli*), and a strain of fungi: *Candida albicans*, ATCC 10,231 (*C. albicans*) from Microbial Collection of ICECHIM, Romania.

*Diffusion method through spot inoculation*

The evaluation of the antimicrobial activity of 10HA-90KNN-CSLG, 90HA-10KNN-CSLG, CSLG (hydrogels), and 10HA-90KNN-CSL, 90HA-10KNN-CSL, CSL (porous scaffolds) was assessed by using the diffusion method through spot inoculation. The tests were performed on a specific agar-based medium: Muller Hinton medium for bacterial strains and Sabouraud medium for *C. albicans*. The agar plate surface was inoculated with microbial suspension by tracking the swab over the entire plate surface. As inoculum, a suspension was used in the sterile physiological water made from a fresh culture of 18–24 h (4–5 isolated colonies) developed on a Tryptone Soy Agar (TSA) medium, with a density of 1–3 × 10^8^ CFU/mL adjusted nephelometrically (McFarland standard 0.5 = 1.5 × 10^8^ UFC/mL). The tested hydrogels were added in a volume of 100 µL, in a spot. Subsequently, the plates were incubated for 24 h at 37 °C in the case of *S. aureus*, and *E. coli* and at 28 °C in the case of *C. albicans*. The tested spongy samples were added in a dry square shape scaffold of 10 mm × 10 mm × 3 mm. Subsequently, the plates were incubated for 24 h at 37 °C in the case of *S. aureus*, and *E. coli* and at 28 °C in the case of *C. albicans*. The antimicrobial activity was evaluated by measuring the diameter of the clear area (halo) appearing around the inoculation area. In addition, the lack of microbial growth under spongy samples can be classified as a strong antimicrobial effect.

The composition of the culture media was as follows: Muller Hinton agar from Scharlau (17.5 g/L peptone; 1.5 g/L starch; 2 g/L meat extract; 15 g/L agar; pH 7.3 ± 0.1 at 25 °C). Tryptic Soy Agar—TSA (15 g/L casein; 5 g/L peptone; 5 g/L NaCl; 15 g/L agar; pH 7.3 ± 0.1 at 25 °C). Sabouraud agar (10 g/L mycological peptone; 40 g/L glucose; 15 g/L agar; pH 5.6 ± 0.2 at 25 °C), physiologic sterile water (8.5 g/L NaCl, 1000 mL distilled water).

## 3. Results and Discussion

### 3.1. Compositional Characterization

#### 3.1.1. XRD

**Figure 1:**
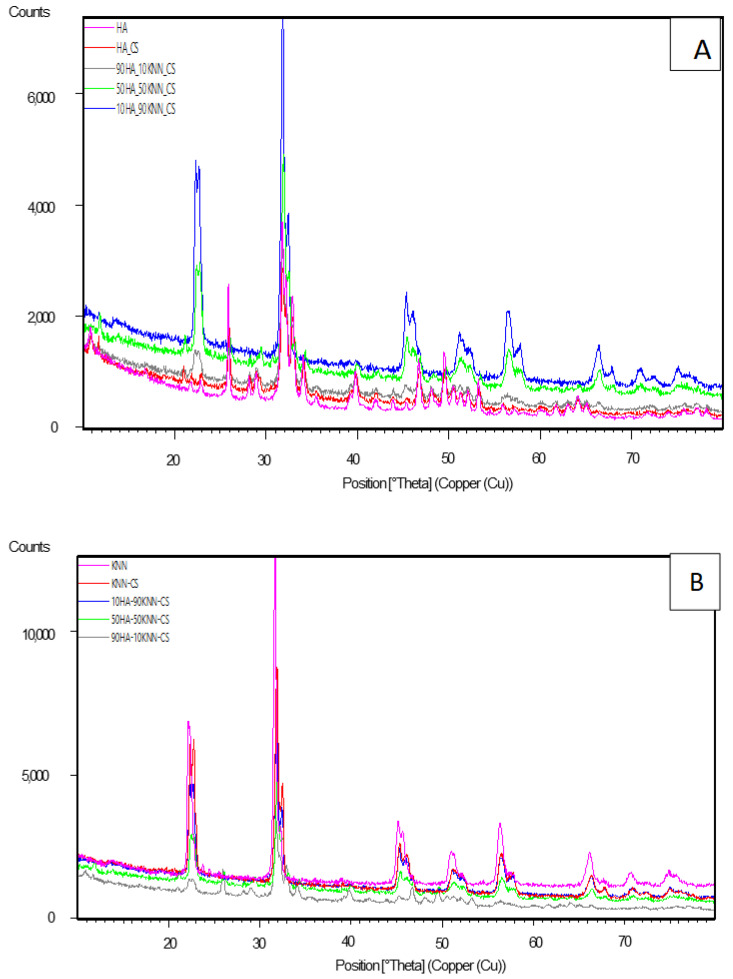
Comparative X-ray diffraction plots of composite scaffolds (10HA-90KNN-CSL, 90HA-10KNN-CSL, 50HA-50KNN-CSL, HA-CSL, and KNN-CSL) and pure powders of HA (**A**) and KNN (**B**).

Comparative X-ray diffraction analysis of porous composite scaffolds (10HA-90KNN-CSL, 90HA-10KNN-CSL, and 50HA-50KNN-CSL) and pure component phases (HA and KNN) are presented in Figure 1A,B. It is evident that the diffractograms for the composites richer in HA (90HA-10KNN-CSL, HA-CSL) resemble the pattern for nanometric HA powder and KNN patterns (10HA-90KNN-CSL, KNN- CSL), respectively. Differences consist of larger peak amplitudes for the two powders, the amorphous phase of chitosan being responsible for the peak’s intensities decrease. These results confirmed the fact that there are no chemical interactions between the HA, CS, and KNN components of the composites, which would determine the formation of undesired secondary phases with specific and different diffraction angles.

#### 3.1.2. FTIR

**Figure 2:**
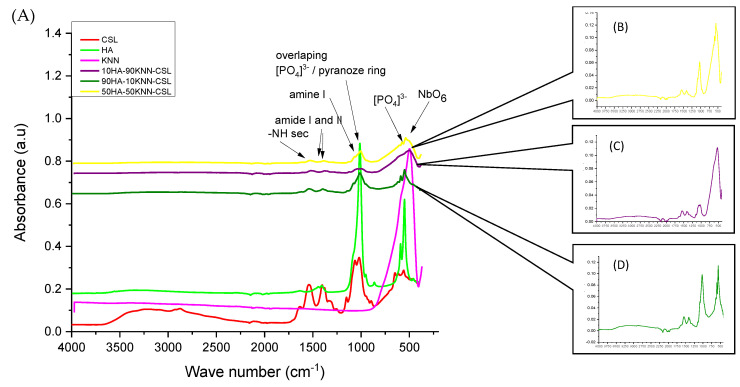
(**A**) Comparative FTIR spectra for porous composite scaffolds 10HA-90KNN-CSL, 90HA-10KNN-CSL, 50HA-50KNN-CSL, and HA, KNN patterns; (**B**) FTIR spectrum for 50HA-50KNN-CSL; (**C**) FTIR spectrum for 10HA-90KNN-CSL; (**D**) FTIR spectrum for 90HA-10KNN-CSL.

The FTIR spectra (Figure 2A) of the composites 90HA-10KNN-CSL, 10HA-90KNN-CSL, and 50HA-50KNN-CSL were compared to those of the initial constituent (pure) phases (HA, KNN, and CSL). The intensity of the absorption peaks decreases progressively for the composites, proportionally with the decrease in pure content. These changes can be attributed to ionic nature interactions between the protonated amino groups and the nanometric HA particles through the phosphate groups, as suggested by some authors [60]. The characteristic bond vibrations between the atoms of functional groups overlap each other, leading to the broadening of absorption peaks such as: from HA the PO_4_^3−^ group at 1060–1020 cm^−1^ with amine groups or from CS or respectively CO_3_^2−^ over amide (-CN and C=O) at 1650–1320 cm^−1^ [61] and from KNN the bond vibration O-Nb-O in NbO_6_ octahedra or PO_4_^3−^ with those of pyranose heterocyclic saturated chains of chitosan in the range of wave numbers ~530 cm^−1^ and 1000–1100 cm^−1^. The amino and amido functional groups of CS positioned between 1655–1340 cm^−1^ remain sufficiently intense, which could provide the significant antibacterial and hemostatic effect of the three composites. Additionally, it is confirmed that no undesired secondary phases occurred between the three basic compounds of composite (HA, KNN, and CS).

### 3.2. Morpho-Structural Characterization

#### 3.2.1. SEM Micrography

**Figure 3:**
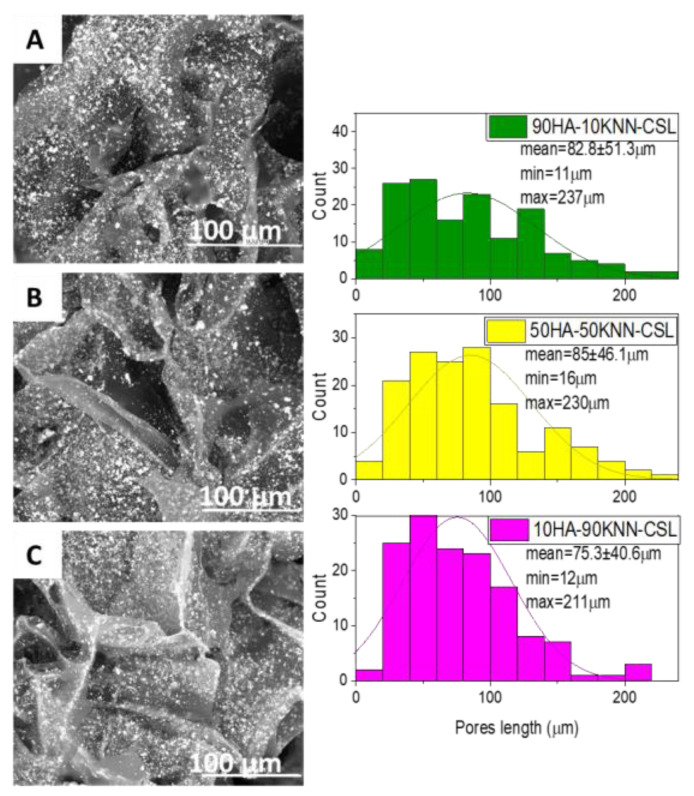
SEM images of lyophilized composite scaffolds: 90HA-10KNN-CSL (**A**), 50HA-50KNN-CSL (**B**), 10HA-90KNN-CSL (**C**). Histograms with the pore length distribution in 10HA-90KNN-CSL, 50HA-50KNN-CSL, and 90HA-10KNN-CSL sponges.

From the morpho-structural point of view, the three scaffolds are almost identical, as can be seen in Figure 3A–C and Figure S1A–C Supplementary data. All three samples contained the same quantity of CSL and different proportions of HA and KNN in the powder mixture (10%, 50%, and 90%) that did not influence the texture and pore sizes. It is observed that the porosity is irregular in distribution and size, and most of the pores are oblong (Figure 3). The pores of the three compositions were macro, as was measured in the SEM images: 70% of the pores’ lengths were <100 μm, the mean length around 80μm and the range between 11 and 237 μm for all samples (Figure 3 histogram).

#### 3.2.2. BET Specific Surface Area

The BET specific surface area was determined on porous grains of 10HA-90KNN-CSL, 50HA-50KNN-CSL, and 90HA-10KNN-CSL samples prepared by grinding the cryogenized corresponding scaffolds.

**Figure 4:**
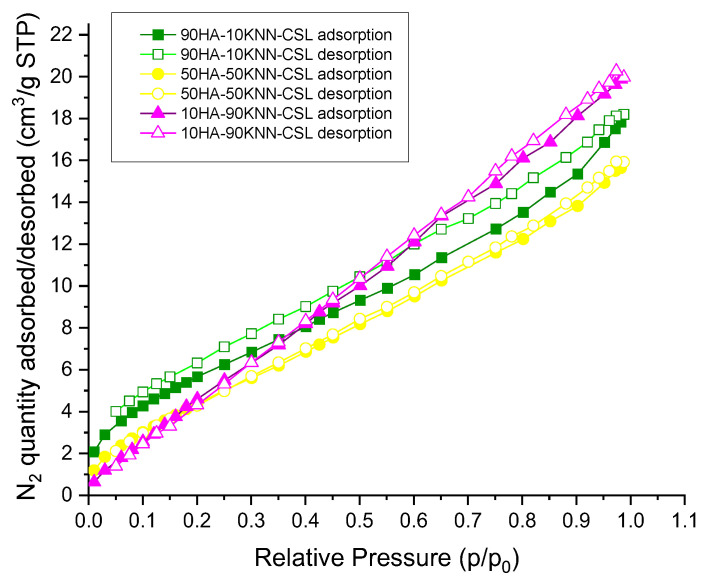
N_2_ adsorption–desorption isotherms for 10HA-90KNN-CSL, 50HA-50KNN-CSL, and 90HA-10KNN-CSL.

Adsorption–desorption isotherms shapes could be associated with micro and meso- porous absorbents (type I and II, Figure 4). The high porosity of the scaffolds observed in SEM images is confirmed by the large BET specific surface area (18.4–24 m^2^/g), the highest value is obtained for the sample 90HA-10KNN-CSL that contained 90% HA in the powder mix (Table 2). Apparently, in the composition 90HA-10KNN-CSL, the mesoporous nanoparticles of HA, having a greater specific surface area, are responsible for the higher value of scaffold surface area. The sample 50HA-50KNN-CSL with 50% HA followed a lower value (21.8 m^2^/g). In connection with that, only for this sample (90HA-10KNN-CSL), a hysteresis loop could be observed (Figure 4, green), the gas quantity adsorbed being higher than the desorbed one.

Additionally, in Table 2 it can be observed that for all three composites grains, the mean diameters are largely placed in the field of macropores (>5 nm); the maximum pores sizes are <0.2 μm and minimum sizes less than 2 nm. Such grand porosity of the composite scaffolds can give improved wettability and intimate contact with water, biomolecules, and cells from the physiological environment.

The SEM images of the 10HA-90KNN-CSL, 50HA-50KNN-CSL, and 90HA-10KNN-CSL grain samples thus prepared are shown in Figure S2A–C Supplementary data. The same irregular distribution and oblong shapes of pores are highlighted. As could be observed (Figure S3A–D Supplementary data), the composite grains with 90% HA had an average size of 66.5 ± 39.7 μm and pores sizes < 200 nm, while the sample 90HA-10KNN-CSL had the larger grains with a mean size of 120 ± 67.5 μm and a gaussian pore size distribution with mean size 265 ± 170 nm. The results from SEM images concerning pore sizes measured on both grains and scaffolds are in accordance, taking into account the consistent standard deviations involved; using this technique it was impossible to detect mesopores detected by the BET method. On the other hand, the pore size differences detected after comparison between the two methods increased because the BET method involved the assimilation of the granule’s shape with spherical ones, and this is far from reality. Nonetheless, the exact knowledge of the pore sizes has no mandatory importance for the functional properties of the composites.

### 3.3. Swelling Test in SBF

**Figure 5:**
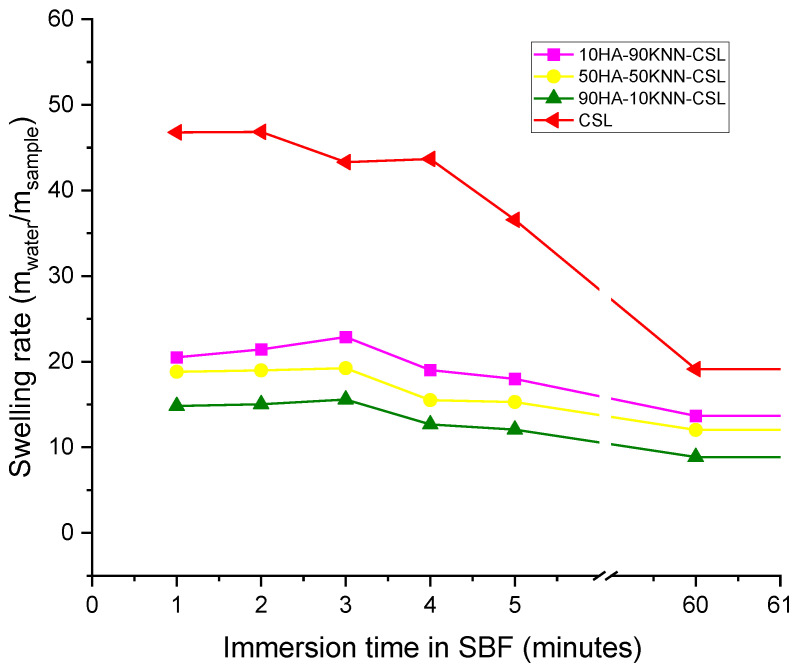
Swelling rate of composite sponges for different immersion times.

The importance of accurately preparing an SBF solution that reproduces the composition of the physiological fluid lies in the success of simulating the biomaterials’ degradation in in-vitro conditions [59,62,63,64]. Figure 5 shows that all tested scaffolds can absorb up to 23 times more SBF than the dry samples mass. Over time, the behavior of all three composite scaffolds is similar in the first 5 min, the highest SBF quantity absorbed was observed at 10HA-90KNN-CSL. The chitosan CSL sponge can retain an amount of liquid around 50 times higher than the corresponding dry mass of the scaffold. The composite scaffolds are stable to fragmentation in the immersed solution for 60 min without attempting to extract them from the immersion medium. Nevertheless, this short stability of scaffolds in contact with SBF was a desired behavior that led to fast clot formation during the grafting operation in the alveolar cavity.

These results show the great wettability of the three composites, which opens the premises of in vivo intimate contact and interaction with body fluid.

### 3.4. Degradation of HA-KNN-CSL Series Sponges in SBF

The grafting composites were designed to be bioactive materials, meaning the chitosan matrix should be easily swelled to induce clot formation and antibacterial effect and also to have a moderate capacity of degradation, at least in SBF, that offered the possibility of releasing the aceto-glycans oligomers, constituents of bone extracellular matrix. Additionally, freeing the HA nanocrystals from the CS matrix, an important Ca^2+^ source that activated the osteoinductive phenomenon, and KNN micrometric perovskite crystals that acted as osteopromotor agents, are desirable processes.

**Figure 6:**
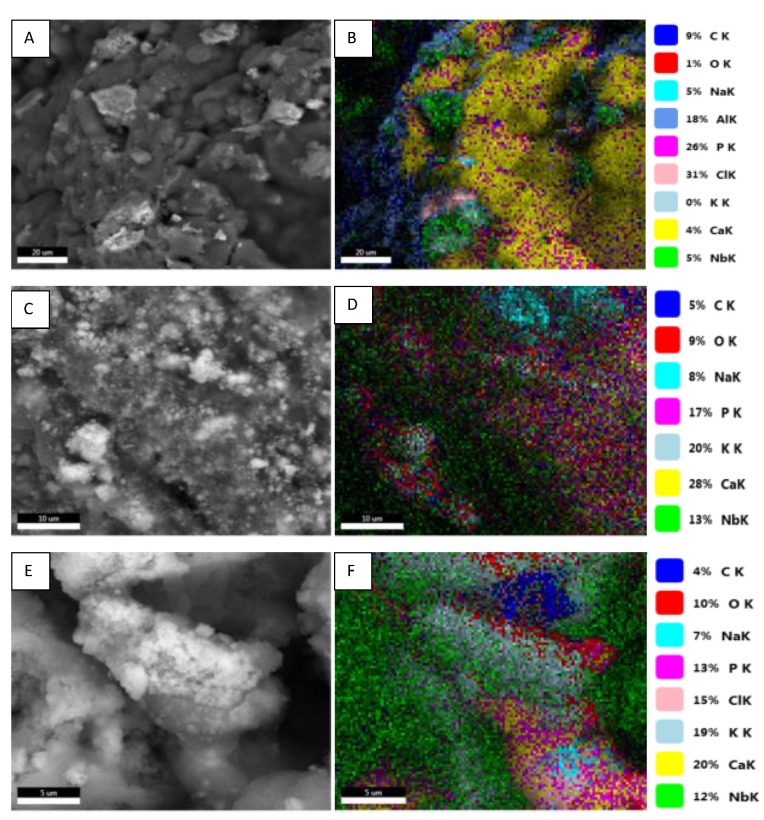
SEM images (**A**,**B**) 10HA-90KNN-CSL (bar-20 μm), (**C**,**D**) 50HA-50KNN-CSL (bar-10 μm), and (**E**,**F**) 90HA-10KNN-CSL (bar-5 μm), EDS elemental mapping and quantitative elemental compositions for scaffolds after 7 days’ incubation in SBF and slowly dried at 23.5 °C for 24 h.

SEM images in Figure 6A,C,E show the morpho-structural changes of 10HA-90KNN-CSL, 50HA-50KNN-CSL, and 90HA-10KNN-CSL scaffolds after 7 days of incubation. The HA and KNN powder particles embedded in the chitosan matrix are exposed but still captive and the porous texture was replaced with a denser one because of biopolymer degradation. Furthermore, the EDS mapping (Figure 6B,D,F) highlighted elemental distribution accordingly with the specific composition on the surface of the analyzed samples: K, Na, and Nb from KNN, Ca, P, and O from HA and C, O from CS. It detected the presence of chlorine in high concentrations, which came from the deposition or crystallization of salts from SBF that had electrostatically interacted, mainly with the cationic nature of the chitosan matrix.

**Figure 7:**
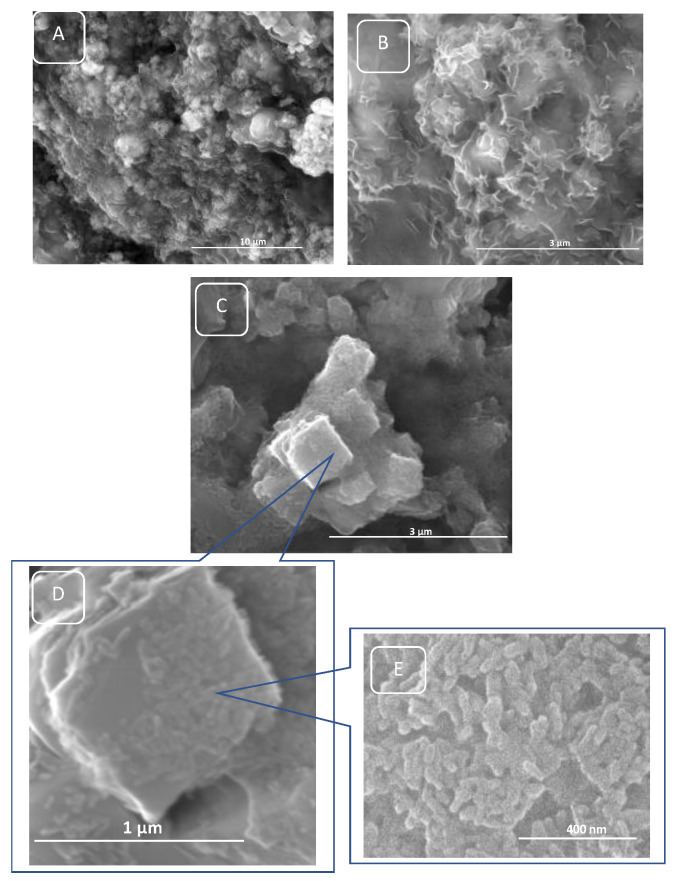
SEM images of the sponge after 28 days of incubation followed by slow drying at 23.5 °C for 24 h: (**A**,**B**) 50HA-50KNN-CLS and (**C**–**E**) 10HA- 90KNN-CLS.

The degradation of the polymer matrix (CSL) of porous scaffolds progressed after 28 days as can be observed in Figure 7A–E. Chemical degradation of the polysaccharide chains was not complete (Figure 7A,B), the HA and KNN granules are exposed from the degraded chitosan matrix (Figure 7C–E). As observed (Figure 7D,E), embedded nanorods of HA are strongly attached to cubic KNN particles and keep the same morphology because of their chemical strength in these neutral pH conditions.

The evaluation of the elemental quantitative results from EDS analyses (Table 3) highlighted that the high percentage of carbon before incubation decreased dramatically after 28 days of incubations in SBF. The hydrolysis reactions of glycoside bonds of CS polysaccharide chains occurred in the SBF medium at 37 °C. These reaction products were oligosaccharides and aliphatic salts, some of them leaving the scaffolds and solubilizing in an aqueous solution. In addition, the exposed HA particles remained chemically unchanged after 28 days, proof that the C/P rate was around 1.67 hydroxyapatite specific value. Chlorine concentration on the treated scaffold increased after 28 days as a result of chlorides crystalizing on the scanned surfaces.

**Figure 8:**
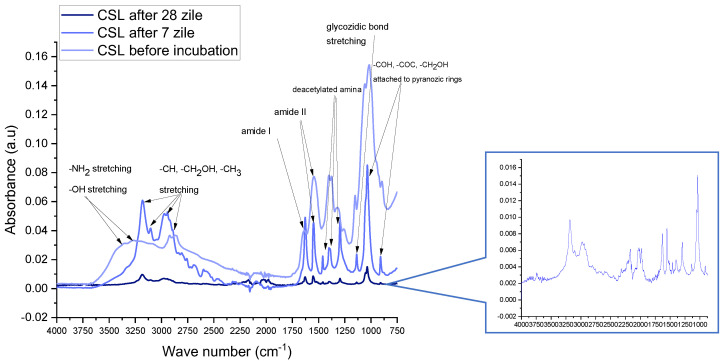
Comparative FTIR absorption spectra of chitosan sponges before and after 7 and 28 days of incubation in SBF.

The best proof of chitosan degradation in SBF was provided by FTIR analyses (Figure 8). Overlapped vibrations stretching mode of -NH2 and -OH bonds at 3353 cm^−1^ and 3271 cm^−1^ in CSL were observed to be more intense before incubation, and decreased after 7 and 28 days, being replaced by stronger vibrations at 3182, 3106, 2976, and 2942 cm^−1^ generated by asymmetric and symmetric stretching mode of aliphatic radicals (-CH, -CH_2_OH, -CH_3_) detached from saccharide rings. The small shoulder at 1631 cm^−1^ became an individual sharp peak marking the increase of the deacetylation process by stretching of the carbonyl bond in the amide I and bending of the -NH bond attached by acetyl groups. Also, individual, high, and sharp peaks at 1402 and 1300 cm^−1^ marked higher vibrations of C-N and -CH_3_ involved in acetyl groups. The shoulder at 1151 cm^−1^ also became sharper and higher because of glycosidic bond hydrolysis and pyranose ring separation. Intense and sharp peaks at 1042 and 913 cm^−1^ represent the stretching of C chains (-COH, -COC, -CH_2_OH) in pyranose rings. Those degradation reactions gradually progress between 7 and 28 days, the two corresponding FTIR spectra showing similar compositional changes but with decreased amplitude (Figure 8, insert).

**Figure 9:**
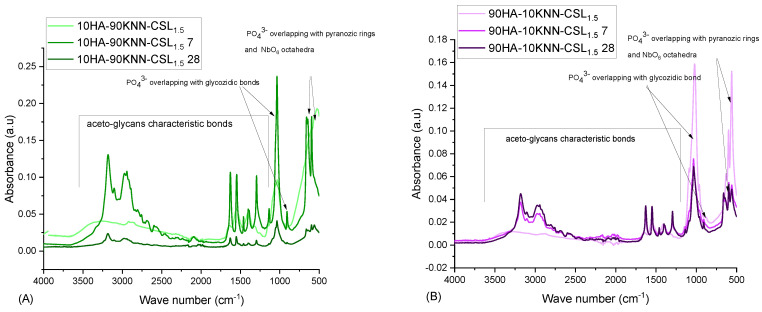
Comparative FTIR absorption spectra of composite sponges before and after 7 and 28 days of incubation in SBF (**A**) 90HA-10KNN-CSL and (**B**)10HA-90KNN-CSL.

The porous scaffolds of 10HA-90KNN-CSL and 90HA-10KNN-CSL comparative FTIR spectra are illustrated in Figure 9A,B. Similar findings are highlighted in these two images: before incubation, the FTIR spectra of the composite scaffolds present absorption bands characteristic to aceto-glycans bonds of CS, as well as absorption bands determined by PO_4_^3−^ group vibrations of HA and O-Nb-O bond vibrations (NbO_6_ octahedra) of KNN. After 7 and 28 days, these last peaks remained sharpened and tall, showing the chemical stability of HA and KNN in SBF conditions incubation. The major changes were observed in the range of characteristic functional groups of CS as described.

### 3.5. In Vitro Biocompatibility of 10HA-90KNN-CSL and 90HA-10KNN-CSL Scaffolds and Hydrogels

**Figure 10:**
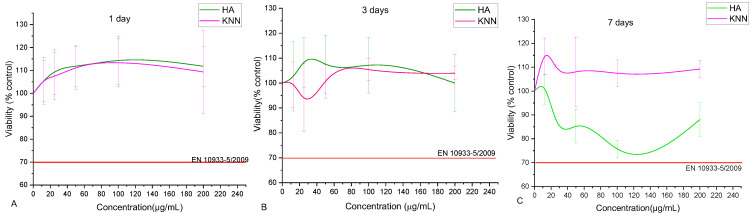
Osteoblast-like MG-63 relative viability (MTT assay after 1 (**A**), 3 (**B**), and 7 (**C**) days of incubation) in the presence of different concentrations (12.5–200 µg/mL) of HA and KNN powders.

In the context of biomaterials, the biocompatibility of HA powder has been previously extensively demonstrated. However, because KNN is also a component of the proposed composites, testing its biological behavior is mandatory, especially since KNN is not a natural component of bone tissue. The osteopromotor activation mechanism also depends on the polarization degree of the piezoelectric material, i.e., by the electric charge generated on the surfaces of the polarized KNN granules. In our case, having no method for external electric polarization of the KNN powder, we relied on an unquantifiable method of powder “self-polarization” by grinding in the stage that preceded the incorporating one into the biocomposite. Hence, the two powder components of composites, HA and KNN, were tested in contact with osteoblast-like MG-63 cells using the MTT tetrazolium salt-based viability assay, which evaluates the metabolism ability of the cells. According to EN ISO 10993-5, the results obtained for both samples at all investigated concentrations (between 12.5–200 µg/mL) are biocompatible. Similar reports have been previously made, showing that submicron KNN powders induced osteoblast MG-63 activation, resembling HA nanometric powders’ effects [32,65,66]. Increasing concentrations of HA and KNN induced superior metabolic activity of osteoblasts, up to 113% (for 100 µg/mL); subsequently, a slight capping trend is recorded (109% at 200 µg/mL). After three days of contact incubation, the relative viability induced by the powders slightly decreased, and the osteoblasts obtained a physiological level of metabolic activity for all tested concentrations (Figure 10B). Unexpectedly, after 7 days of incubation, all concentrations of KNN powder induced higher metabolic activity, while the increasingly higher HA powder concentrations seemed to induce a higher oxidative stress on MG-63 cells, almost to the limit of biological incompatibility (Figure 10C). Therefore, it could be assumed that the KNN powder induces no cytotoxic effect against the osteoblast MG-63, and the “self-polarization” of piezoelectric particles leads to metabolic cell activation. However, the data was not statistically significant compared to control samples.

**Figure 11:**
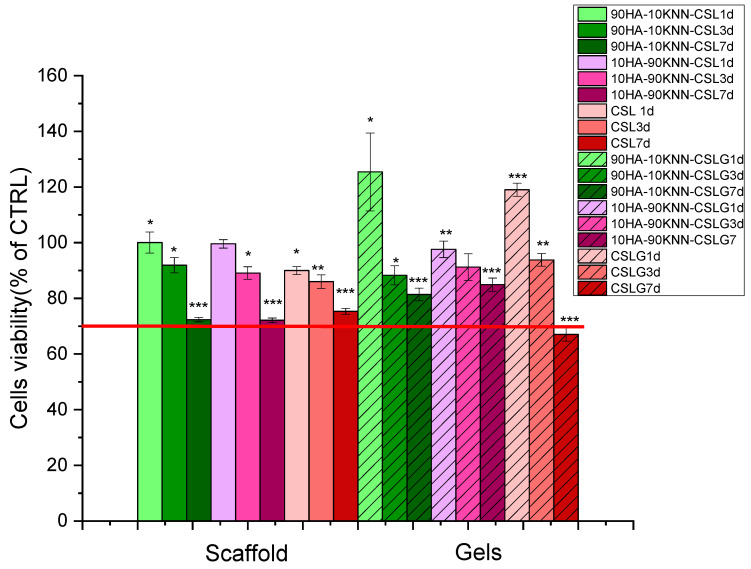
Osteoblast-like MG-63 relative viability (MTT assay after 1, 3, and 7 days in the presence of 90HA-10KNN-CSL, 10HA-90KNN-CSL, and CSL dry sponges and 90HA-10KNN-CSLG, 10HA-90KNN-CSLG, and CSLG gels; the statistical analysis: two-tailed Student’s test * *p ≤* 0.05, ** *p ≤* 0.01, *** *p ≤* 0.001 were considered as statistically significant.

Additionally, the viability of the osteoblast-like cells in contact with both scaffolds and gels was determined using the MTT test following 1, 3, and 7 days of contact incubation (Figure 11).

As evidenced in Figure 11, all tested samples (scaffolds and gels) induced cellular viability higher than 70% of CTRL, which qualifies them as biocompatible materials according to the standard “EN ISO 10993-5:2009 Tests for in vitro cytotoxicity” specifications. A lower cell metabolic activity was induced by the scaffolds, in comparison to gel samples (Figure 11), as a result of improved cell-sample interactions in the gel form. The two scaffold-type compositions (90HA-10KNN-CSL and 10HA-90KNN-CSL) showed an almost physiological metabolic cell activity (viability about 100%, compared to control, NS) after one day of incubation. Sponges and gels with the highest HA content (90HA-10KNN-CSL and 90HA-10KNN-CSL G) induced the highest cell metabolic activation (100.5%, NS and 125.4%, *p* < 0.05, compared to control) after the first day of contact, followed by a descending trend of around 90% of CTRL after three days of incubation and lower results (>70% of CTRL) but still within biocompatibility limits—for 7 days. Similarly, the composition with 90% KNN powder content (10HA-90KNN-CSL and 10HA-90KNN-CSLG) determined a high activity of osteoblast MG-63, both in scaffold and hydrogel form (99.6%, NS and 119%, *p ≤* 0.01, respectively) after the first day of incubation and the same descendent trends highlighted after 3 and 7 days of contact incubation. However, after three days of incubation, the gel sample (10HA-90KNN-CSLG) exhibited the highest viability compared to the other two jelly samples. Such behavior could be assumed by piezoelectric component contribution which demonstrated similar biocompatibility as well-known hydroxyapatite in contact with the osteoblasts’ culture medium. Moreover, the gamma radiation used for KNN powder sterilization could have the polarization effect of piezoelectric grains that induces the cell’s viability increase, as reported in the case of ultrasound irradiation [67]. The CSL sponge and CSLG hydrogel induced the scantiest oxidoreductase enzyme amount secreted by osteoblasts, in the presence of samples, by comparison with composites samples for all incubation periods. The MTT assay utilized for osteoblast-like viability quantification in contact with powder HA and KNN, as well as composite gels and scaffolds, highlighted that the piezoelectric powder is not only biocompatible, but it has the potential to improve the metabolic activity of bone-forming specialized cells, even under minimal conditions of external stimulation, such as mastication.

**Figure 12:**
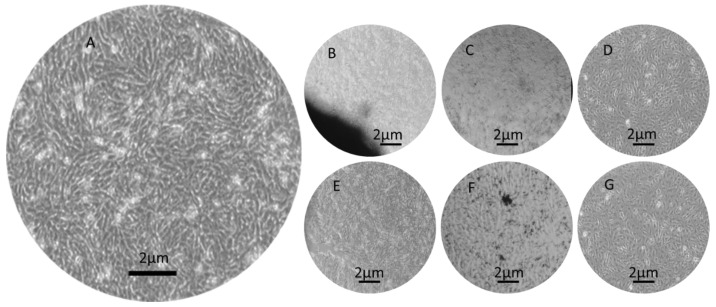
Optical microscopy images (5×) of samples after one day of incubation in contact with MG-63 osteoblast-like: (**A**) negative control (CTRL), (**B**) scaffold 90HA-10KNN-CSL, (**C**) scaffold 10HA-90KNN-CSL, (**D**) scaffold CSL, (**E**) hydrogel 90HA-10KNN-CSLG, (**F**) hydrogel 10HA-90KNN-CSLG, (**G**) hydrogel CSLG.

The cell morphology in contact with composite scaffolds (Figure 12B–G) was not severely affected after one day of incubation compared to control samples (Figure 12A). In the case of gel samples, HA and KNN nanoparticles were released in the cell culture medium and their presence was evidenced as dark aggregates in the cell monolayer (Figure 12F). Increasing the incubation time at 7 days, the optical micrography evidenced elongated osteoblasts with shapes similar as in control (untreated) samples. The cellular density decreased in the case of hydrogel samples as compared to the negative control (Figure S4 Supplementary data). These results were in concordance with viability measurements.

### 3.6. Antibacterial and Antifungal Activity of 10HA-90KNN-CSL and 90HA-10KNN-CSL Hydrogels and Scaffolds

**Figure 13:**
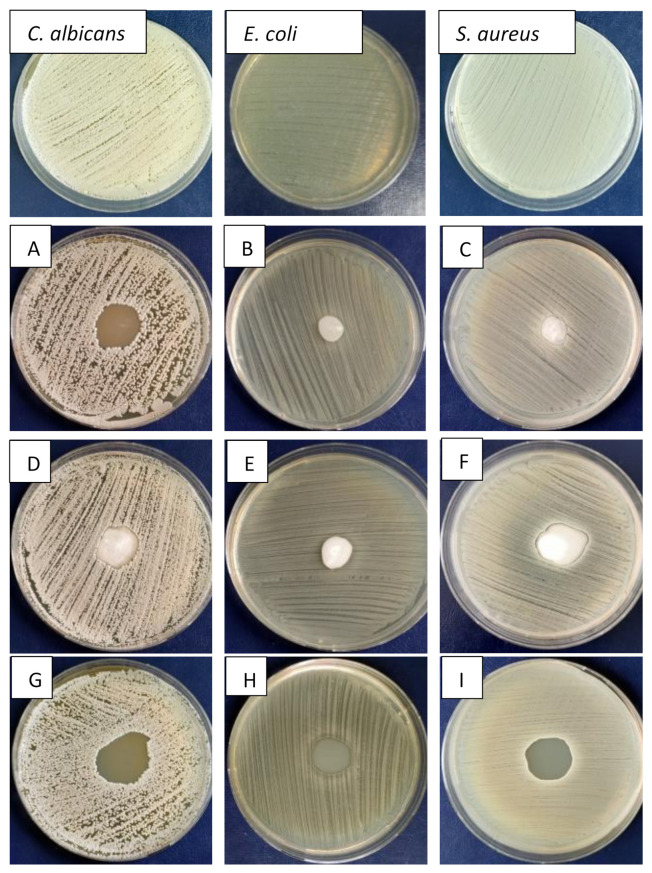
Antibacterial (*E. coli* and *S. aureus*) and antifungal (*C. albicans*) effects of hydrogels spots by disc diffusion method: (**A**–**C**) 90HA-10KNN-CSL G (avers), (**D**–**F**) 10HA-90KNN-CSL G (avers), and (**G**,**H**) CSL G (**I**).

The antibacterial/fungal activity of synthesized hydrogels 90HA-10KNN-CSLG, 10HA-90KNN-CSLG, and CSLG was evaluated by spot disc diffusion method against *E. coli*, *S. aureus*, and *C. albicans* using the protocol described in Section 2.2.7. The three strains were considered representative bacterial and fungal strains in the oral cavity. Therefore, the effect of bacterial and fungal plates in contact with spots of different composites gel samples in the same amount (100 μL) was shown in Figure 13A–I.

**Figure 14:**
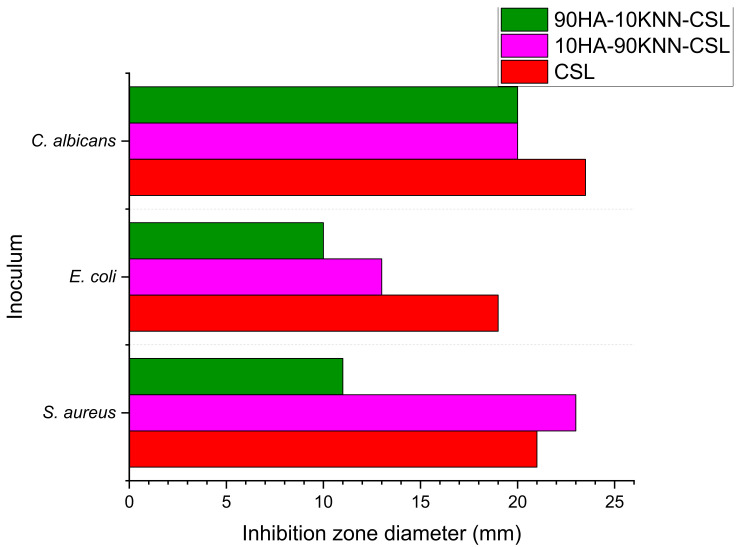
The inhibition zone diameters by disc diffusion in spot method of 10HA-90KNN-CSLG, 90HA-10KNN-CSLG, and CSLG hydrogels on the *S. aureus*, *E. coli*, and *C. albicans* inoculum.

The images of the inhibition zones measurement of the three gels (90HA-10KNN-CSLG, 10HA-90KNN-CSLG, and CSLG) were presented in Figure 14. Due to the well-known antimicrobial effect and the richer content of chitosan (6 mg/100 μL), the sample CSL G had the most extensive inhibition zone diameters against *C. albicans* (23.5 mm) and smaller (19 mm) in the case of *E. coli* (Figure 14). The sample 10HA-90KNN-CSLG, which contains only 2 mg/100 μL CS and 1.26 mg/100 μL of KNN, showed improved antimicrobial activity, especially against gram-positive bacteria *S. aureus* with the inhibition zone diameter of 23 mm, wider than CSLG (21 mm), and a weaker effect against the two other strains: *C. albicans* and *E. coli* (20 mm and 13 mm). An important antibacterial effect was reported of piezoelectric polarized powders or scaffolds; therefore, this desired antimicrobial behavior of KNN powder was highlighted [10,42,68]. As expected, the last positions went to 90HA-10KNN-CSLG, caused by the lowest chitosan and KNN content, the increased HA amount in composite (1.26 mg/100 μL) that boosted microbial metabolic activity, which decreased the inhibition zone diameters compared with the other two gels, for all tested strains (*C. albicans* 20 mm, *S. aureus* 11 mm, and *E. coli* 10 mm). Compared with CS (CSLG sample) which could be considered a renowned antimicrobial agent, as expected, the sample 10HA-90KNN-CSLG seems to show a decreased effect against gram-negative bacteria (*E. coli*). As reported, the negative charge of poled KNN particles sides caused the slowing down of the bacterial membrane—biomaterial grains interactions [15,54].

**Figure 15:**
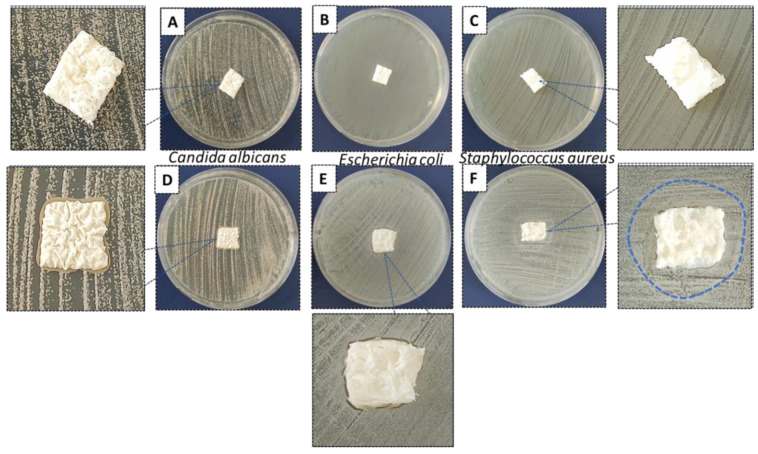
Antibacterial (*E. coli* and *S. aureus*) and antifungal (*C. albicans*) effects of dry scaffolds by disc diffusion method: (**A**–**C**) 90HA-10KNN-CSL and (**D**–**F**) 10HA-90KNN-CSL.

Samples 90HA-10KNN-CSL and 10HA-90KNN-CSL in the form of dry lyophilized scaffolds were also tested, as described in the protocol in Section 2.2.7, on the same three strains: *S. aureus*, *E. coli*, and *C. albicans*. The images regarding the antimicrobial behavior of the square-shaped samples inoculated, without prior wetting, on the surface of the bacterial and fungal culture media in the agar plates, are shown in Figure 15. After 24 h of contact between dry scaffolds and inoculum, regardless of its type, the composition with 90% HA (90HA-10KNN-CSL) did not interact with the bacterial/fungal culture media. Moreover, these scaffolds remained dry. However, for the samples 10HA-90KNN-CSL with the highest content of piezoelectric powder, the formation of a slightly opaque halo can be observed with a diameter of 26.4 mm, proof of interaction with *S. aureus*, a clear area below and along the scaffold perimeter, and which it exceeded by approximately 1 mm in contact with *E. coli* plate and a 1 mm thick darker cord along the perimeter of the scaffold, marking an interaction of the composite with the *C. albicans*.

The antibacterial/antifungal effect was intensively increased with the more reactive gel form of the two compositions compared with corresponding dry scaffolds, for 24 h of inoculant contact, and the strongest effect of 10HA-90KNN-CSL was especially highlighted against gram-positive bacteria (*S. aureus*). Nonetheless, the effect of gamma radiation used for composite scaffold sterilization did not show boosted antimicrobial activity, as is reported for hydroxyapatite and chitosan-based biomaterials [69].

## 4. Conclusions

Biocomposites in the compositional system HA-KNN-CSL were synthesized by varying the proportions of HA and KNN powders in the form of hydrogels and porous scaffolds. These (nano)components were chosen to bring functional properties for bone substitute application in dentistry: compositional mimicry of bone, biocompatibility, excellent SBF absorption, adhesion, and wettability due to high porosity, antibacterial and antifungal features, and potential osteopromotor effects as a result of piezoelectric properties and versatility in application forms. The results showed that the 90% KNN samples induced the best antimicrobial effect, as well as high osteoblast-like cell metabolic activity, in the absence of external stimulation. The samples in the gel form revealed a lower biological reactivity compared to scaffolds. Moreover, due to the acetic acid solubilization of chitosan, the biocomposites can degrade in SBF following 28 days of incubation, even without enzyme addition. The synthesized compositions proved to be biocompatible and showed antibacterial effects. They could have prospective applications as bone grafts in dentistry for cases in which the piezoelectric component of the composite could be activated during mastication, and could lead to shorter bone healing duration; however, further in vivo evaluation is necessary.

## Figures and Tables

**Table 1:** Sample denomination and detailed composition for 2 g of HA-KNN-CS biocomposite.

Sample Name	Hydroxyapatite (HA) (g)	Potassium–Sodium Niobate (KNN) (g)	Chitosan * (CSL) (mL)	Type
CSLG	-	-	30 mL	gel
90HA-10KNN-CSLG	1.26	0.14	30 mL	gel
10HA-90KNN-CSLG	0.14	1.26	30 mL	gel
50HA-50KNN-CSLG	0.7	0.7	30 mL	gel
CSL	-	-	30 mL	scaffold
90HA-10KNN-CSL	1.26	0.14	30 mL	scaffold
10HA-90KNN-CSL	0.14	1.26	30 mL	scaffold
50HA-50KNN-CSL	0.7	0.7	30 mL	scaffold

* 2% Chitosan with low molecular density, 1.5% acetic acid aqueous solution.

**Table 2:** BET specific surface area and pore diameters (mean, maximum, and minimum sizes) variation at adsorption–desorption for 10HA-90KNN-CSL, 50HA-50KNN-CSL, and 90HA-10KNN-CSL samples.

Property	10HA-90KNN-CSL	50HA-50KNN-CSL	90HA-10KNN-CSL
BET specific surface area ± SD (m^2^/g)	18.4 ± 0.7	21.8 ± 0.4	24 ± 1.9
Mean pore diameter—adsorption (4 V/A) ± SD (nm)	20.8 ± 34.8	19.7 ± 33.7	19.7 ± 33.5
Mean pore diameter—desorption (4 V/A) ± SD (nm)	12.5 ± 15.6	12 ± 15.3	11.7 ± 15
Maximum pore diameter (adsorption nm)	161.2	74.6	154.6
Minimum pore diameter (adsorption nm)	1.8	1.6	1.7

**Table 3:** EDS elemental quantitative compositions of 10HA-90KNN-CSL, 50HA-50KNN-CSL, 90HA-10KNN-CSL scaffolds before and after 28 days of incubation in SBF.

Identified Element	10HA-90KNN-CSL		50HA-50KNN-CSL		90HA-10KNN-CSL	
	Quantity (at. %/abs. Error %)					
	Before	After	Before	After	Before	After
**C K**	48.4/99.9	9.9/99.9	47.1/90.9	15.2/99.9	32.6/99.9	11.2/99.9
**O K**	14.4/10.6	8.5/13.8	26.2/21	20.5/13	41/13.8	24.6/10.6
**Na K**	1.1/9.3	35/8.6	1.8/1.3	20.8/7.4	2/18	19.8/9.3
**P K**	0.9/7.7	3.1/16.2	1.6/0.15	6.6/18.2	7.7/22.6	7.7/7.7
**Nb L**	18.6/36.2	10.2/21.7	17.5/22.9	4/22.9	7.6/21.7	-
**Cl K**	13.3/6.6	27.5/5.6	-	22.2/4.1	-	14.4/6.6
**K K**	1.7/5.5	0.5/22.6	3/22.3	0.2/29.2	1.9/5.1	9.5/5.5
**Ca K**	1.5/15.7	5.2/18.1	2.8/7.3	10.5/73.3	7.1/82.3	12.9/22.7
**Ca/P**	1.63	1.67	1.78	1.60	0.92	1.68

## Data Availability

Not applicable.

## Supplementary Materials

The following supporting information can be downloaded at: https://www.mdpi.com/article/10.3390/polym15112446/s1, Figure S1: SEM images of lyophilized composite scaffolds: 90HA-10KNN-CSL (A), 50HA-50KNN-CSL (B), 10HA-90KNN-CSL (C); Figure S2: SEM images of composite grains obtained from porous scaffold cryogrinding: 90HA-10KNN-CSL (A), 50HA-50KNN-CSL (B), 10HA-90KNN-CSL (C); Figure S3: Histograms of the composite grains 10HA-90KNN-CSL(A), 50HA-50KNN-CSL(B) and 90HA-10KNN-CSL(C) and the comparative histograms of the grains pores size distribution(D); Figure S4: Optical microscopy images (5x) of samples after 7 days of incubation in contact with osteoblast-like MG-63: (A) negative control(CTRL), (B) scaffold 90HA-10KNN-CSL, (C) scaffold 10HA-90KNN-CSL, (D) scaffold CSL, (E) hydrogel 90HA-10KNN-CSLG, (F) hydrogel 10HA-90KNN-CSLG, (G) hydrogel CSLG.
