# Supplementary material for: Piezoelectric Biocomposites for Bone Grafting in Dentistry
Source: Polymers (Basel). 2023 May 25;15(11):2446. doi: 10.3390/polym15112446 (PMC10255500; doi:10.3390/polym15112446)
Supplement: Supplementary file 1 [file polymers-15-02446-s001.zip › polymers-2324809-supplementary.pdf]

SUPPLEMENTARY DATA:

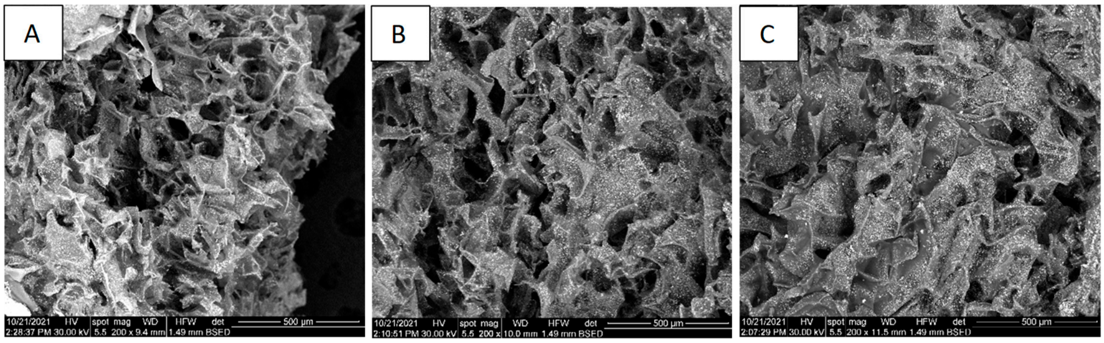

Figure S1. SEM images of lyophilized composite scaffolds: 90HA-10KNN-CSL (A), 50HA-50KNN-CSL (B), 10HA-90KNN-CSL (C)

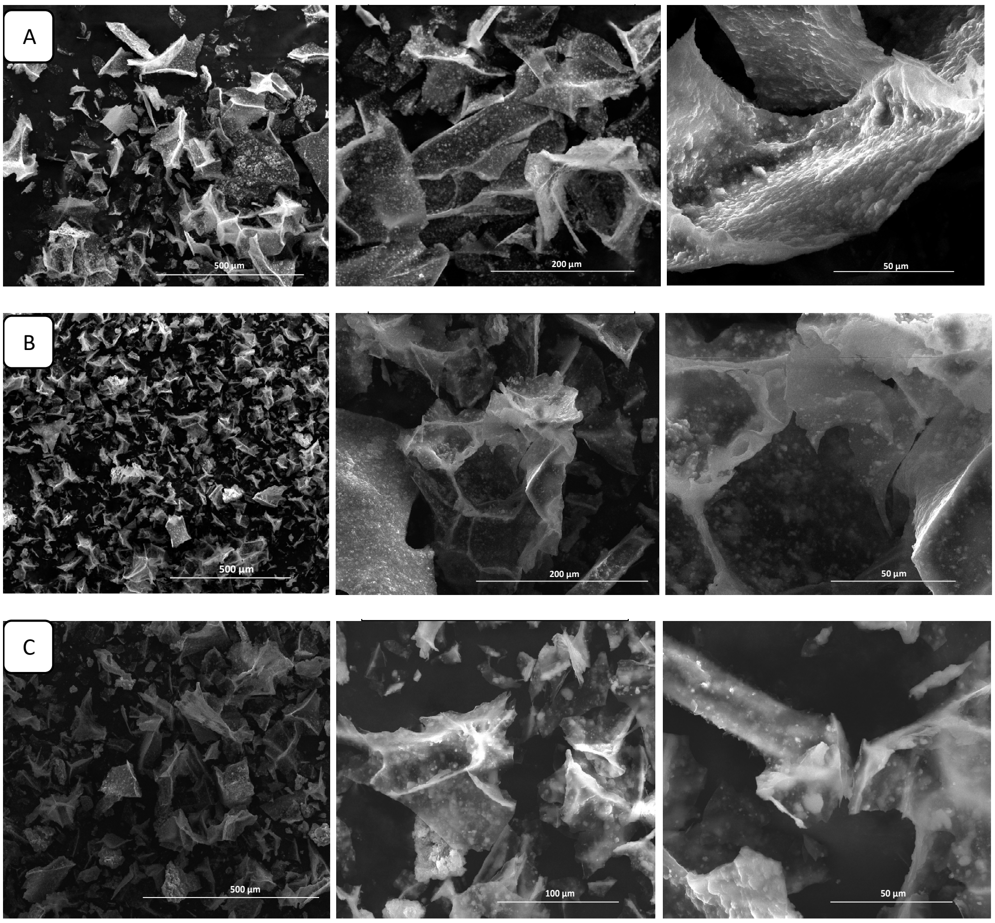

Figure S2. SEM images of composite grains obtained from porous scaffold cryogrinding: 90HA-10KNN-CSL (A), 50HA-50KNN-CSL (B), 10HA-90KNN-CSL (C)

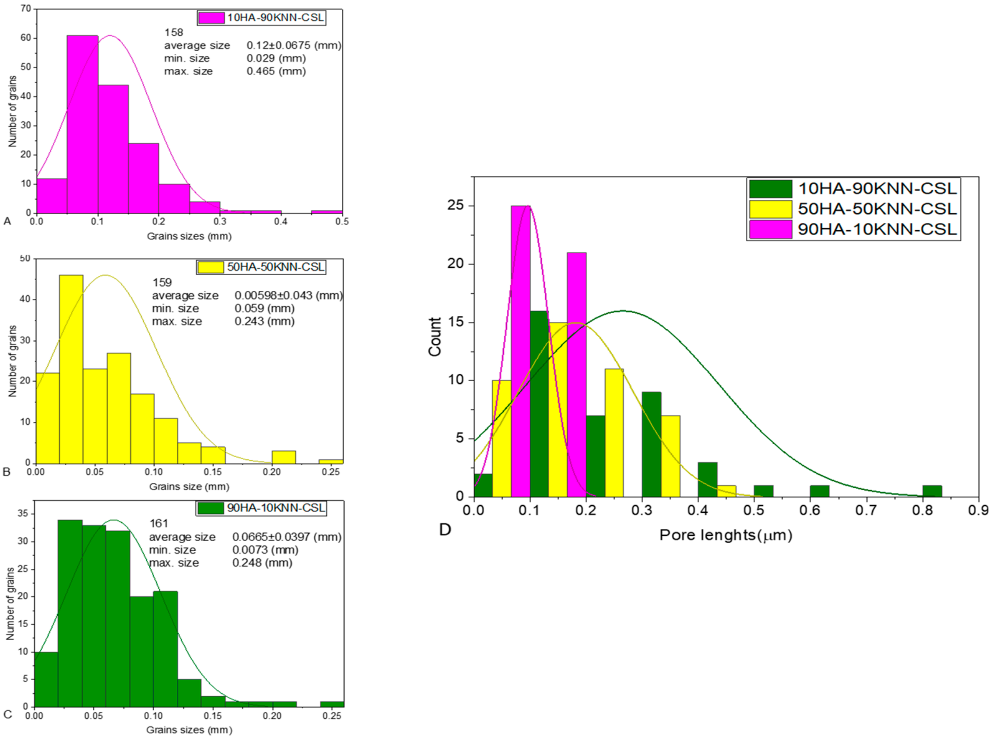

Figure S3. Histograms of the composite grains 10HA-90KNN-CSL(A), 50HA-50KNN-CSL(B) and 90HA-10KNN-CSL(C) and the comparative histograms of the grains pores size distribution(D)

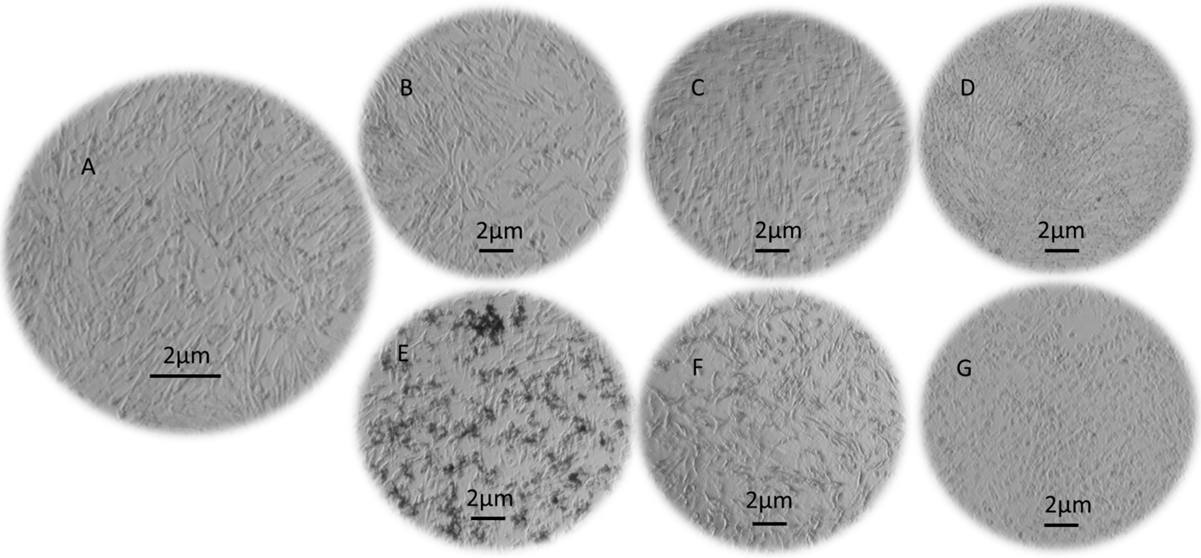

Figure S4. Optical microscopy images (5x) of samples after 7 days of incubation in contact with osteoblast-like MG-63: (A) negative control(CTRL), (B) scaffold 90HA-10KNN-CSL, (C) scaffold 10HA-90KNN-CSL, (D) scaffold CSL, (E) hydrogel 90HA-10KNN-CSLG, (F) hydrogel 10HA-90KNN-CSLG, (G) hydrogel CSLG.
